# Reconstructing Pleistocene Australian herbivore megafauna diet using calcium and strontium isotopes

**DOI:** 10.1098/rsos.230991

**Published:** 2023-11-22

**Authors:** Dafne Koutamanis, Matthew McCurry, Theo Tacail, Anthony Dosseto

**Affiliations:** ^1^ Wollongong Isotope Geochronology Laboratory, School of Earth, Atmospheric and Life Sciences, University of Wollongong, Wollongong, New South Wales 2522, Australia; ^2^ Centre for Archaeological Science, School of Earth, Atmospheric and Life Sciences, University of Wollongong, Wollongong, New South Wales 2522, Australia; ^3^ Australian Museum Research Institute, Sydney, New South Wales, Australia; ^4^ Earth and Sustainability Science Research Centre, School of Biological, Earth and Environmental Sciences, University of New South Wales, Kensington, New South Wales 2052, Australia; ^5^ Paleobiology, NMNH, Smithsonian Institution, Washington, DC 20560, USA; ^6^ Institute of Geosciences, Johannes Gutenberg University, Mainz, Germany

**Keywords:** megafauna, marsupials, trophic level, weaning, calcium isotopes, strontium isotopes

## Abstract

Isotopes in fossil tooth enamel provide robust tools for reconstructing food webs, which have been understudied in Australian megafauna. To delineate the isotopic composition of primary consumers and understand dietary behaviour at the base of the food web, we investigate calcium (Ca) and strontium (Sr) isotope compositions of Pleistocene marsupial herbivores from Wellington Caves and Bingara (New South Wales, Australia). Sr isotopes suggest small home ranges across giant and smaller marsupial herbivores. Ca isotopes in Pleistocene marsupial herbivores cover the same range as those in modern wombats and placental herbivores. Early forming teeth are depleted in heavy Ca isotopes compared to late-forming teeth of a given individual, suggesting a weaning signal. Distinct Ca compositions between taxa can be interpreted as dietary niches. Some niches conform to previous dietary reconstructions of taxa, while others provide new insights into niche differentiation across Australian herbivores. Combined with the small roaming ranges suggested by Sr isotopes, the Ca isotope niche diversity suggests rich ecosystems, supporting a diversity of taxa with various diets in a small area.

## 1. Introduction

During the Pleistocene, Australia was inhabited by a diverse assortment of large-bodied mammals, reptiles, and birds that are referred to as the Australian megafauna. These now-extinct animals would have greatly influenced past ecosystems with bottom-up and top-down pressures caused by actors in food webs that do not exist today [1]. It is fundamental to reconstruct feeding relationships in these food webs in order to understand the influences that these species had on the environment and on each other, and to know how Pleistocene ecosystems operated. Feeding relationships play important roles in hypotheses on the cause of megafauna extinction events, including overhunting of juvenile prey by humans (e.g. [2]) and increased resource competition in response to climate change (e.g. [3,4]). Yet, the reconstructions of feeding relationships required to understand the complexity of interactions between these extinct animals, and potentially with humans, have received little attention [5,6]. This is largely owing to the lack of a trophic level proxy suitable for Pleistocene fossils.

Isotopic systems allow for the observation of nutrient flows through ecosystems; consequently, isotope ratios in bioapatite (the mineralized component, hydroxyapatite, of teeth and bones) of an individual reflect various ecological conditions and dietary behaviours during its life [7]. Carbon (C) isotope ratios (δ^13^C) in bioapatite reflect the consumed vegetation type (i.e. C_3_ or C_4_ plants) and can be extrapolated to distinguish browsers from grazers (e.g. [8]). Oxygen (O) isotope ratios (δ^18^O) in bioapatite are dependent on the source of drinking water and can inform about climate and in the individual's habitat (e.g. [9]). Strontium (Sr) isotope ratios (^87^Sr/^86^Sr) vary according to geological substrate of an organism's water and food resources and can be used to trace mobility of individuals in landscapes [10]. While Sr isotope analysis is widely used in palaeontology, it has only been applied to a single megafauna specimen from Australia [11].

So far, C and O isotopes in Australian fossil fauna show niche partitioning among co-existing herbivorous taxa throughout time [5,6] and dietary shifts among macropods in response to environmental change during Pleistocene climatic fluctuations [9,12]. Sequential δ^13^C and δ^18^O values in continuously growing *Diprotodon* incisors show seasonal dietary fluctuations; in a single individual, additional sequential ^87^Sr/^86^Sr ratios link this seasonal diet to migratory behaviour [5,11]. While these ecological proxies have been effective for dietary reconstructions and relationships among herbivores, they do not reveal the dietary dynamics of the greater faunal community. Early geochemical analyses, including trace element ratios, and C and N isotopes, on Australian megafauna included a trophic level reconstruction of *Thylacoleo carnifex*, indicating a carnivorous diet [9,13]. Early geochemical analyses were prone to diagenetic alteration of biological signals in skeletal remains which prevented wider application to Pleistocene fossils from Australia.

Calcium (Ca) isotopes can be analysed in dental enamel, where the high-density, crystalline structure minimizes the possibility of diagenetic overprint [14–16]. Furthermore, as its main component, the high concentration of Ca in bioapatite (approx. 40 wt. %), in contrast with low concentrations in geological materials, result in minimal diagenetic alteration of biogenic Ca. The Ca isotope composition (expressed as δ^44/42^Ca) of bioapatite reflect diet and trophic levels in both terrestrial and marine ecological communities owing to preferential uptake of light ^42^Ca over heavy ^44^Ca isotopes during bioapatite formation and therefore a consistent change in Ca isotope composition between food and consumers [14,16–21]. Moreover, Ca isotope compositions differ between herbivore foraging behaviours (i.e. browsing and grazing) within the same ecosystem. This may be owing to Ca isotope fractionation occurring between roots and leaves of dicotyledons—the dominant component of a browsing diet—which is not observable in monocotyledons, i.e. grasses—the main food resource of grazers [18,20,22–24]. In addition, Ca isotopes can indicate nursing and weaning behaviour in placental mammals by comparing the ratios in enamel of teeth that formed early in life during milk consumption (owing to the light Ca isotope composition of milk) to enamel of teeth of the same individual that formed during adulthood [25–29]. The combination of Sr and Ca isotopes can provide a spatial component to dietary reconstructions, resource distribution and the geographical extent of impact of megafauna on the ecosystem as predators or ecological engineers. However, Sr isotopes have not yet been explored in combination with Ca isotopes in fossil teeth of Pleistocene Australian fauna.

The application of Ca isotopes to modern Tasmanian bare-nosed wombats showed that the Ca isotopic signature of this Australian marsupial opportunistic grazer covers the same range of values as African placental herbivores [30]. High-resolution sequential Ca isotope data in combination with δ^13^C and δ^13^O values in wombat teeth indicate that Ca isotopes could identify seasonal changes in the types of vegetation consumed [30,31], as dicots and monocots are characterized by different Ca isotope compositions [18,22]. Higher Ca isotope values observed in wombat females suggest the same potential as in placental mammals for Ca isotopes to identify lactation in marsupials [30], as bioapatite from lactating female placentals has also been recorded to have higher isotopes compositions (e.g. [28]). Thus, Ca isotopes can examine many aspects of dietary behaviour in the food web; yet they have not been applied to Australian Pleistocene taxa.

Prior to using Ca isotope analysis for the reconstruction of unknown food web relationships in extinct Australian megafauna, it is essential to document the Ca isotope composition of the primary consumers. A baseline consisting of dietary behaviour of megaherbivores can provide insight into the bottom-up pressures that influenced Pleistocene Australian ecosystems. Furthermore, the characterization of the Ca isotopic composition in varying herbivore diets can capture the range of Ca isotope values in food resources available to predators.

**Figure 1.**
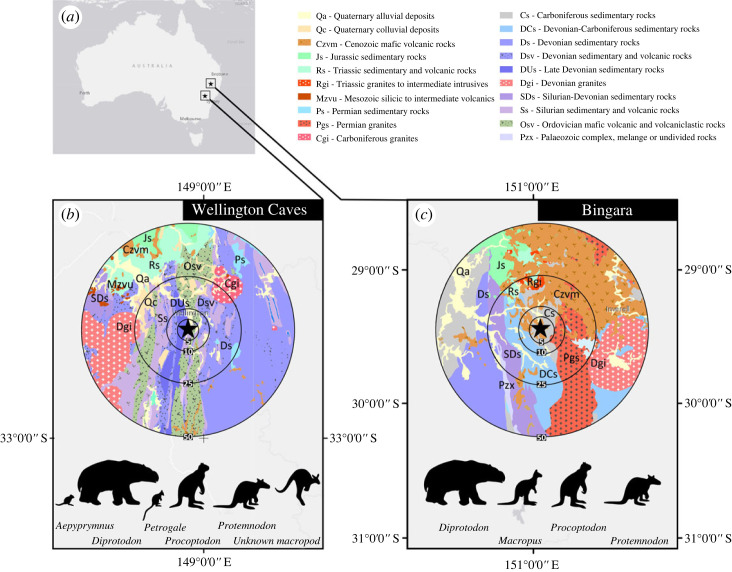
Location and geological substrate (derived from [66]) of the study areas. Panel (*a*) shows location of sites in Australia. Panels (*b*) and (*c*) show the local geological maps within a 50 km distance from the sites. Numbers in the circles refer to radius in km. Silhouettes illustrate the fossil taxa included in this study for each site. Taxonomic symbols were modified from www.phylopic.org.

Here we apply Ca isotope analysis combined with Sr isotope analysis on fossil remains from Australian marsupial herbivores of various body sizes from Wellington Caves and the Bingara region (northeast New South Wales, Australia; figure 1). The aims of this study are:

— to assess the home ranges in Pleistocene Australian fauna using Sr isotopes;
— to characterize the Ca isotope composition of Australian mega marsupial herbivores and explore how it relates to different herbivorous diets; and
— to investigate whether the Ca isotope values in marsupial teeth that form early in life reflect the consumption of maternal milk.

## 2. Material and methods

### 2.1. Materials

Fossil dental remains of Pleistocene herbivorous marsupials were obtained from the Australian Museum Palaeontology collection. The term megafauna is used in this study to refer to the Australian Pleistocene fauna that include large-bodied taxa but does not denote any specific size class of animal here, as we aimed to sample a wide range of sizes and feeding ecologies of Pleistocene herbivores. The samples were acquired from 57 teeth of 37 individuals (electronic supplementary material, table S1) from two sites: Wellington Caves, on Wiradjuri Country, and Bingara, on Kamilaroi Country, New South Wales. Taxa include *Aepyprymnus* (*Aepyprymnus rufescens*; rufous bettong; *n* = 5), *Diprotodon* (*Diprotodon australis*, *Diprotodon optatum*, *Diprotodon* sp; ‘giant wombat’; *n* = 6), *Macropus*, (*Macropus cf. rufus*, red kangaroo; *n* = 5), *Petrogale* (*Petrogale* sp., rock wallaby; *n* = 6), *Procoptodon* (*Procoptodon goliah, ‘*giant kangaroo’; *n*= 5), *Protemnodon* (*Protemnodon* sp., ‘giant wallaby’, *n* = 4), and one unknown genus of the Macropodidae family (referred to as ‘unknown macropod’ in this study; *n* = 6).

#### 2.1.1. Study area: geology and expected strontium bioavailability

The samples derived from historical specimens collected in a non-systematic manner; therefore, little is known about their chronological and depositional context. No Sr isotope compositions are known from any geological units in either region (i.e. that of Wellington Caves and Bingara). However, for both sites, the regional geological substrate and surroundings can suggest expected ranges for ^87^Sr/^86^Sr ratios in the landscape.

The Wellington Caves complex is situated in a river valley on a massive lime-mudstone substrate of the Middle Devonian Garra Formation [32]. The complex consists of multiple, possibly interconnected caves that contain various deposits with fossil fauna. A general stratigraphy consists of three units: bedrock is overlain with the Phosphate Mine beds, containing Pliocene fossil fauna, and subsequently the Mitchell beds, a bone cave breccia unit of Pleistocene age [32,33]. The specimens in the present study most likely originate from the Mitchell beds. Fossil accumulation in this unit is still poorly understood but may be the result of natural pit-fall traps [33]. The fauna from the Wellington Cave complex probably roamed on the limestone-mudstone and adjacent volcanic and sedimentary rocks and overlying alluvial sediments of the river valley (see figure 1). Devonian limestone is predicted to have a ^87^Sr/^86^Sr ratio between 0.708 and 0.709: a typical seawater signal for the Devonian [34]. Volcanic rocks in the region can be expected to have a relatively low ^87^Sr/^86^Sr ratio, between 0.705 and 0.712, while the Sr isotope signal of alluvial sediments and sedimentary rock will depend on the original rocks but, on average, are likely to have a higher ^87^Sr/^86^Sr ratio than young volcanic rocks [10]. Older granites to the west and northeast of the Wellington Cave complex (as illustrated in figure 1) will contribute a higher ^87^Sr/^86^Sr ratio (typically higher than 0.710) to the bioavailable Sr in the region [10,34–36].

Bingara is located in northeast New South Wales and is part of the New England Orogen, a Palaeozoic tectonic fold belt [37]. The Bingara region is mostly underlain by Devonian, Carboniferous, and Permian deposits with intrusions of granite and metamorphic rock [38]. Tertiary uplift and subsequent basalt erosion and downcutting created valley deposits consisting of clays, gravels and sands, which filled in meandering channels, such as Myall Creek [38,39]. Pleistocene faunal fossils were found embedded in the alluvial deposits of the Myall Creek, within a single grey, sandy claystone horizon [40,41]. Geospatial analysis was conducted to assess the origin of the fossil deposit and is described in the electronic supplementary material (§A and figure S1): the creek's drainage area suggests that the material is likely to have derived from within 1 km of the fossil deposition located on the same substrate consisting of Devonian volcanic (tholeiitic basalt and dolerite) bedrock and volcanoclastic deposits (see the electronic supplementary material, figure S1). While ^87^Sr/^86^Sr ratios of the bedrock are unrecorded, the Tertiary basaltic sedimentary rocks would suggest a ^87^Sr/^86^Sr ratio higher than typical uneroded basalt, i.e. higher than 0.705 [34] and are expected to be within the range of typical for volcanoclastic substrates, i.e. below 0.712. In addition, the bioavailable Sr may have influences of higher ^87^Sr/^86^Sr ratios where older granites extrude, the latter of which tend to be higher than 0.710 [10,34,36].

#### 2.1.2. Background: marsupial dentition and life history

Absolute timing of tooth formation in both extant and extinct taxa studied here is unknown; yet, among mammals the sequence of relative timing of enamel mineralization roughly follows the sequence of tooth eruption (electronic supplementary material). While considerable variation in the timing of lactation, tooth eruption and mineralization can occur across marsupial taxa, M1 and M2 generally form before the young first exits the pouch and the weaning process starts, i.e. introduction to non-milk foods. By contrast, M3, M4 and I3 are usually fully erupted by the time weaning has been completed [42–44]. This pattern has been confirmed by previous isotopic studies that showed variation between early-, and late-forming teeth in modern macropods, suggesting nursing and weaning signals [12,45]. Based on the tooth development and weaning behaviour in extant marsupials that are closely related to the taxa in this study (see the electronic supplementary material for more details), we consider M1 and M2 ‘early-forming teeth’, while ‘late-forming teeth’ refers to M3, M4, and I3. Because of its variable timing in eruption and long tooth growth, P3 is considered intermediate in the sequence of tooth formation and eruption.

### 2.2. Analytical techniques

For each sample, the tooth type, taxonomy, and location were recorded (electronic supplementary material, tables S1, S3–S5). For each individual, at least one late-forming tooth was sampled, i.e. M3, M4, or I3. Where possible, multiple teeth per individual, or two locations on an isolated tooth were sampled, where one sample was taken close to the crown of the tooth, and one close to the root of the tooth. The crown is considered to contain the earliest enamel deposition, while the enamel near the root contains the latest enamel depositions [43,46–48]. As the exact trajectory of the growth axis may vary across tooth types and taxa, sampling at these two locations was the most secure approach to circumvent any such variation [43,46–48]. Well-preserved dental enamel was targeted and collected using a rotary tool with a diamond-coated or tungsten steel drill bit (see the electronic supplementary material for details).

Samples were prepared for Sr and Ca isotope analyses in a Class 10 cleanroom at the Wollongong Isotope Geochronology Laboratory (WIGL), University of Wollongong, and analysed on a Thermo Scientific Neptune Plus multi-collector inductively coupled plasma mass spectrometry, as previously described [30]—see the electronic supplementary material for more details.

Ca isotope results were calculated relative to an in-house standard (Alfa Aesar Specpure Ca plasma standard), expressed as *δ*^44/42^Ca_WIGL_, defined as

2.1 $$\delta^{44/42}\mathrm{Ca}{}_{\mathrm{WIGL}}=\;\left(\frac{{{(}^{44}\mathrm{Ca}{/}^{42}\mathrm{Ca})}_{\mathrm{sample}}}{{{(}^{44}\mathrm{Ca}{/}^{42}\mathrm{Ca})}_{\mathrm{WIGL}}}-\;1\;\right)*\;1000,$$

where (^44^Ca/^42^Ca)_WIGL_ is the ^44^Ca/^42^Ca ratio of the primary standard. The δ^44/42^Ca_WIGL_ data were converted to δ^44/42^Ca_SRM915a_ (i.e. relative to isotopic reference material NIST SRM 915a) by adding 0.527‰ in order to allow comparison with published data from other studies. Conversion to data expressed against other primary standards and to δ^44/40^Ca values can be found in the electronic supplementary material, table S2. To express a difference between the δ^44/42^Ca composition of early- and late-forming teeth, we use the following notation:

2.2 $$\Delta^{44/42}{\mathrm{Ca}}_{\mathrm{early}-\mathrm{late}}=\delta^{44/42}{\mathrm{Ca}}_{\mathrm{early}}-\;\delta^{44/42}{\mathrm{Ca}}_{\mathrm{late}},$$

where ‘early’ and ‘late’ refer to early-forming and late-forming teeth, respectively, of a given individual. Details on quality control of all analyses, including assessment of blanks, isotopic standards, and mass-dependent fractionation can be found in the electronic supplementary material, §B, figure S2 and table S2).

## 3. Results

### 3.1. Element concentrations

Fossil samples show some enrichment in rare earth elements (REE) compared to modern wombat enamel from Tasmania [30]. However, the concentrations of bioessential trace elements (e.g. Zn, Mn, Fe, Cu and Sr) are within the range of those in modern enamel (electronic supplementary material, §C and figures S2–S3). Only two samples show enrichment across all REE and trace elements; they were excluded from further analyses (electronic supplementary material, §C). The rest of the assemblage shows no correlations between enriched elements and either ^87^Sr/^86^Sr or δ^44/42^Ca_SRM915a_ values (electronic supplementary material, figures S3–S4).

### 3.2. Strontium isotopes

**Figure 2.**
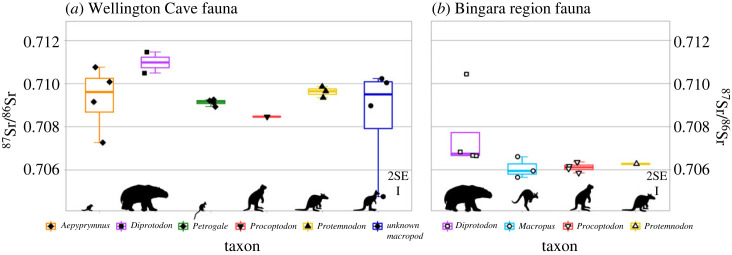
Overview of ^87^Sr/^86^Sr ratios in all individuals. Each symbol is the mean value for aliquots from adult teeth of a given individual. Panels (*a*) and (*b*) display boxplots of ^87^Sr/^86^Sr ratios per taxon at Wellington Cave and Bingara, respectively. Error bars represent the mean 2 s.e. of measurements (see the electronic supplementary material for details).

For a given individual, the mean Sr isotopic composition was calculated from subsamples of the same individual. These mean values of the individuals were used for comparison between and within taxa and sites. Fauna at Wellington Caves (figure 2*a*) shows a larger range of Sr isotope compositions between and within taxa than fauna at Bingara (figure 2*b*). Of all taxa at Wellington Caves, the unknown macropods show the largest variability, followed by *Aepyprymnus*, with mean ^87^Sr/^86^Sr ratios of 0.7085 ± 51 (2 s.d., *n* = 4) and 0.7093 ± 30 (2 s.d., *n* = 4), respectively. *Diprotodon* has a relatively high Sr isotope composition (0.7110 ± 14; 2 s.d., *n* = 2), while *Petrogale* and *Protemnodon* have intermediate mean ^87^Sr/^86^Sr ratios of 0.7091 ± 3 (2 s.d., *n* = 4) and 0.7096 ± 5 (2 s.d., *n* = 3), respectively, and show little inter-individual variability. A single *Procoptodon* individual at Wellington Caves has a relatively low ^87^Sr/^86^Sr ratio of 0.70847 ± 5 (2 s.e., internal analytical uncertainty).

By contrast, the taxa at Bingara show a narrower range of Sr isotope compositions for each taxon (figure 2*b*). *Macropus* and *Procoptodon* have mean ^87^Sr/^86^Sr ratios of 0.7061 ± 10 (2 s.d., *n* = 3) and 0.7061 ± 4 (2 s.d., *n* = 4), respectively. A single *Protemnodon* individual has a similar ^87^Sr/^86^Sr ratio of 0.70627 ± 5 (2 s.e. internal analytical uncertainty). *Diprotodon* have a higher ^87^Sr/^86^Sr ratio compared to other taxa at the same site, with a mean of 0.7076 ± 37 (2 s.d., *n* = 4), including one outlier with a ^87^Sr/^86^Sr ratio of 0.71044 ± 3 (2 s.e. internal analytical uncertainty).

### 3.3. Calcium isotopes in adult individuals

**Figure 3.**
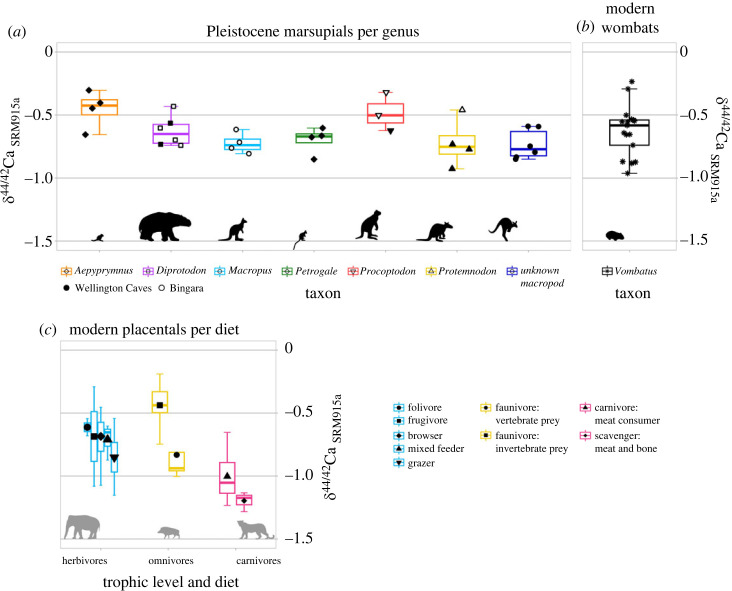
Calcium isotope composition for Pleistocene herbivore marsupials compared to modern mammals. Each symbol is the mean value for aliquots of a given individual. (*a*): Ca isotope composition of Pleistocene taxa in this study. Error bars represent the average 2 s.e. of measurements (see the electronic supplementary material for details). (*b*): Ca isotope composition of modern Tasmanian bare-nosed wombats [30]. (*c*): compilation of previously published data from modern placental mammal dental enamel [18,19,29], converted to δ^44/42^Ca_SRM915a_, grouped by trophic level, and feeding strategy.

The Ca isotope composition of all adult teeth of marsupial herbivores analysed here (mean δ^44/42^ Ca_SRM915a_ = −0.65 ± 0.32‰, 2 s.d., *n* = 31) is similar to that of modern Tasmanian bare-nosed wombats (−0.63 ± 0.40‰, 2 s.d., *n* = 17; [30]; figure 3). There are no statistically significant differences between the mean Ca isotope compositions of each taxon (Kruskal-Wallis, *p* = 0.067; Bonferroni-adjusted *p* > 0.1; electronic supplementary material, table S7). Individuals from Bingara and Wellington Caves have similar Ca isotope compositions with mean δ^44/42^Ca_SRM915a_ values of −0.61 ± 0.31‰ (2 s.d., *n* = 11) and −0.67 ± 0.32‰ (2 s.d., *n* = 20) respectively, with no statistically significant difference between the two sites (Welch two sample *t*-test, *p* = 0.267). Therefore, taxa are considered per genus for the dietary reconstruction of Ca, regardless of their site of origin.

*Aepyprymnus* and *Procoptodon* have the heaviest Ca isotope compositions, with mean δ^44/42^Ca_SRM915a_ values of −0.45 ± 0.30‰ (2 s.d., *n* = 4), and −0.48 ± 0.31‰ (2 s.d., *n* = 3), respectively (see figure 3). These compositions are isotopically heavier than those of placental browsers [18] and at the high end of the range of modern wombats [30]. *Diprotodon* displays an intermediate Ca isotope composition, with a mean of −0.63 ± 0.24‰ (2 s.d., *n* = 6), which is similar to that of modern wombats [30] (figure 3). Lower δ^44/42^Ca_SRM915a_ compositions are observed for *Petrogale* (−0.70 ± 0.21‰, 2 s.d., *n* = 4), the unknown macropod (−0.73 ± 0.23‰ (2 s.d., *n* = 6), *Macropus* (−0.73 ± 0.16‰, 2 s.d., *n* = 4), and *Protemnodon* (−0.72 ± 0.39‰, 2 s.d., *n* = 4) taxa. These values are higher than those of placental African grazers [18] and at the low end of modern wombats [30] (figure 3).

### 3.4. Intra-individual calcium isotope variations

**Figure 4.**
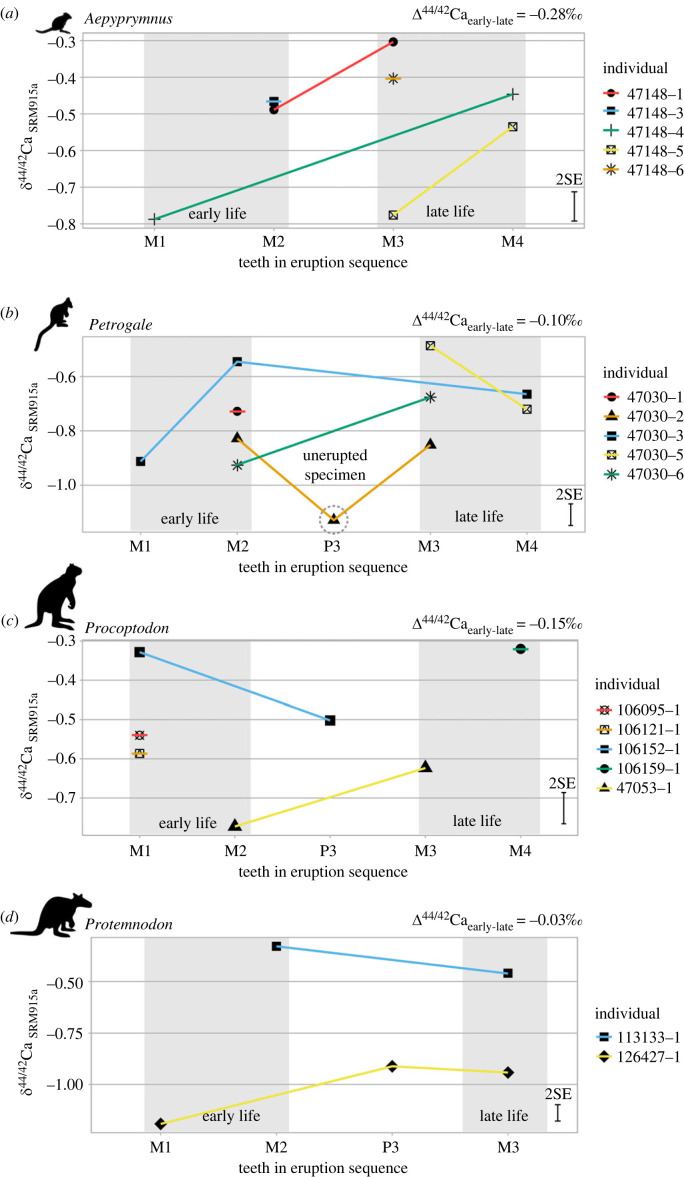
Calcium isotope composition of enamel in various teeth, sorted according to eruptive sequence. Each point is the mean value for aliquots of a given subsample of a single tooth of a given individual. Panels (*a*-*d*) show the Ca isotope compositions of *Aepyprymnus* individuals (*n* = 6), *Petrogale* individuals (*n* = 5), *Procoptodon* individuals (*n* = 5), and *Protemnodon* individuals (*n* = 2), *n* = respectively. Error bars represent the mean 2 s.e. of measurements (see the electronic supplementary material for details). Δ^44/42^Ca_early-late_ given at each panel describes the mean Δ^44/42^Ca_early-late_ for the taxon displayed in said panel.

For individuals for which both early- and late-erupting teeth were available, the difference between the mean value of early-erupting teeth and that of late-erupting teeth of a given individual is expressed as Δ^44/42^Ca_early-late_. All Δ^44/42^Ca_early-late_ values are provided in the electronic supplementary material, table S8 and show a mean of −0.13‰. Mean Δ^44/42^Ca_early-late_ for each taxon are all negative (figure 4), indicating that all taxa display—to some extent—a trend toward increasing *δ*^44/42^Ca_SRM915a_ values with age of enamel mineralization. One notable exception is the low Ca isotope composition of a P3 in *Petrogale* individual 47030-2 (figure 4), which derived from a tooth that had not yet erupted from the jaw and, thus, may have still been in the process of mineralization. Another clear exception is *Procoptodon* individual 106152-1, which—in contrast to the other individuals in the taxon—shows a higher *δ*^44/42^Ca_SRM915a_ value in an early-forming tooth (i.e. M1) compared to that of a tooth from the same jaw, that would have erupted later (i.e. P3).

## 4. Discussion

### 4.1. Diagenetic assessment

A two-step diagenetic screening was conducted to exclude poorly preserved samples from further analyses, as described in the electronic supplementary material. Briefly, preserved biogenic Ca and Sr isotope compositions were indicated by trace and REE within the range of expected modern variation and by lack of correlations between the isotopic values and REE. Sr isotope ratios and Ca isotope compositions in fossil dental enamel of Pleistocene marsupials appear largely unaffected by diagenesis.

### 4.2. Geographical origin and home ranges of Pleistocene megafauna marsupials

For a given site, all taxa show Sr isotope compositions close to expected Sr bioavailability based on the local bedrock (figure 1). The mean ^87^Sr/^86^Sr ratio of 0.70932 in fauna from Wellington Caves is close to the Sr isotope composition of Phanerozoic seawater (approx. 0.709), as would be expected from the Devonian limestone in which the cave complex is situated [10]. Animals roaming on granites less than 25 km away from the cave complex (figure 1) would be expected to have ^87^Sr/^86^Sr ratios higher than 0.710 [10,34–36]. The relatively low Sr isotope composition of Bingara fauna (0.70642) is similar to expected ^87^Sr/^86^Sr ratios of volcanic rocks between 0.704 and 0.706 (e.g. [10]) and thus in agreement with what would be expected for the volcanic to volcanoclastic substrate with influences of sedimentary alluvium at the site's drainage area (electronic supplementary material, figure S1). If animals were roaming on granites less than 10 km to the east of Bingara, ^87^Sr/^86^Sr ratios would be expected to be higher, i.e. higher than 0.710 [10,34–36].

The accordance between ^87^Sr/^86^Sr ratios of fossil teeth and the (assumed) local Sr bioavailability implies, firstly, that the faunal assemblages from both sites are likely to come from geographically constraint areas. Within these sites, no clear spatial partitioning (i.e. distinct roaming ranges) between taxa can be observed. Intra-taxon and intra-individual Sr isotope variation can be explained by roaming on substrates in close proximity to the sites. The high ^87^Sr/^86^Sr ratio of a Bingara *Diprotodon* (figure 2) could be caused by migratory behaviour (e.g. [11]) into areas with Quaternary and Carboniferous sedimentary substrates to the west, within a 25 km radius of the site (figure 1). In the Wellington Cave fauna, a low ^87^Sr/^86^Sr of an *Aepyprymnus* individual (figure 2) might indicate a home range on the volcanic formations that surround the site in the south, north, and southwest within a 10 km distance to the site, rather than the limestone bedrock or the alluvial plain in the east (figure 1). The larger variance in *Aepyprymnus*, compared to other taxa, could be caused by a mixed assemblage of multiple populations. Since there are currently no geochronological constraints on the studied assemblages, specimens may be chronologically distinct.

Modern marsupial home ranges vary with body mass of taxa and environmental conditions [49]. Extant *Aepyprymnus* and *Petrogale* spp. have small home ranges, with recorded maxima of 0.1 km^2^ and 0.2 km^2^, respectively [49]. The recorded home ranges of modern *M. rufus* vary between 0.5 to 21 km^2^ [49,50]. While travel distances usually do not exceed 13 km, changes in vegetation cover have caused populations to shift their home range by travelling 30 km [50,51]. Based on their body masses, the giant macropods *Procoptodon* and *Protemnodon* could be hypothesized to travel further distances than *M. rufus*. A single *Diprotodon* individual has been suggested to have migrated 200 km annually based on Sr isotope analysis [11].

Large home ranges or long-distance migrations in megafauna are not indicated by the Sr isotope compositions observed here for *Diprotodon, Procoptodon*, and *Protemnodon,* as their Sr isotope compositions are consistent with expected Sr isotope ratios of the local bedrock within 10–25 km distance of both sites. Furthermore, their Sr isotope compositions are similar to that of taxa from the same assemblages with smaller body masses. Instead, the ‘local character’ of Sr isotope ratios in the fossil teeth suggests that these extinct megafauna marsupials at the sites studied here could have had a roaming range similar to those of smaller extant marsupials. This hypothesis is supported by observations in modern macropods indicating that climate is a more important determinant of home range size than body mass [49]. The small home ranges observed for a range of taxa both at Wellington Caves and Bingara sites may indicate a rich ecosystem subject to a favourable climate that would have sustained a variety of dietary niches within the same environment, as was recently also suggested for Pleistocene fauna from Mount Etna, Queensland [52].

### 4.3. Calcium isotope compositions of Pleistocene marsupial herbivores

The Ca isotope composition of Pleistocene marsupial herbivores varies between taxa but not for a given taxon across different sites (figure 3). This supports previous findings which showed that Ca isotope compositions of dental enamel are independent of geological substrate [20,30,53]. The Ca isotope composition of Pleistocene marsupial herbivores shows differences between taxa, indicating distinct Ca isotopic niches. These could be reflective of dietary niches, physiological differences (e.g. digestive system), or a combination of the two. The offset between the Ca isotope composition of diet and that of bioapatite appears consistent across terrestrial placental herbivores, irrespective of taxon or digestive physiology (e.g. [18,28]). Furthermore, the digestive physiology of all taxa studied here is expected to be similar to that of modern macropods and potoroids, i.e. foregut fermentation [44]. Thus, although possible digestive differences cannot be excluded, the distribution of Ca isotope compositions observed here is more likely to reflect differences in diet across taxa than in digestive physiology.

*Aepyprymnus* displays high Ca isotope values in the upper quartile of the Ca isotope composition of modern wombats and placental browsers (including frugivores and folivores, figure 3). This could suggest that these *Aepyprymnus* individuals were browsers, which is only partly in agreement with the diet of this extant taxon. Modern rufous bettongs browse on roots, grasses, forbs and fungi [54]; while Ca isotope values from fungi have not been reported, browsing forbs would indeed cause high Ca isotope values, but the consumption of grasses and roots is expected to lower Ca isotope values [55]. The Ca isotope composition of *Macropus* is close to that of modern placental grazers and in the lower quartile of modern Tasmanian wombats, suggesting that these Pleistocene individuals, similar to extant *Macropus*, were also grazers. The Ca isotope composition of *Petrogale* specimens is suggestive of a specialized, monocot-dominated diet: their small range of values covers the lower quartile of modern wombats and is close to that of modern placental grazers. Various modern species in the genus of *Petrogale* show dietary plasticity and feed on mixtures of C_3_ and C_4_ vegetation, and dicot foliage and monocot grasses, varying per species and environment (e.g. [56]). The difference between extant and Pleistocene *Petrogale* diet could be explained by a local adaptation or dietary plasticity through space and time. Indeed, dental microwear and geochemical data in modern and Pleistocene macropods show frequent dietary changes, switching between C_3_ to C_4_ plant-dominated diets throughout environmental changes [5,6,8,9,12,57,58].

*Diprotodon* has a Ca isotope composition within the interquartile range of modern wombats—its closest relative. Previous dental microwear, and C, O, and Sr isotope studies have indicated that *Diprotodon* was an opportunistic generalist with a diet dominated by browsing (C_3_), with seasonal variation in proportions of C_3_ and C_4_ vegetation in tandem with seasonal migratory behaviour [5,11]. Similarities between the Ca isotope composition of *Diprotodon* and modern wombats could suggest similar diets despite different amounts of food required owing to their difference in body mass. *Procoptodon* is characterized by high δ^44/42^Ca values, suggesting a diet of dicot foliage, similar to those encountered in *Aepyprymnus* (figure 3). This is in agreement with a previous dental microwear and C isotopes from previous study, indicating that this taxon has a specialized dietary niche that consisted of foraging a rare C_4_ dicotyledon plant [59]. *Protemnodon* shows δ^44/42^Ca values in the lower quartile of modern wombats (figure 3). Skeletal morphology, dental microwear studies, and C isotope compositions show that *Protemnodon* is a mixed feeder, foraging both C_3_ and C_4_ plants, but with a strong preference for C_3_ vegetation, potentially inhabiting forest environment [5,6]. A mixed diet with a large C_3_ component would be expected to result in high δ^44/42^—Ca values as dicot foliage is enriched in ^44^Ca compared to monocots—[18,22] at odds with our data. The low δ^44/42^Ca values in *Protemnodon* may instead have been caused by specialized consumption of a plant source with a depleted Ca isotopic composition, such as dicot roots and stems. While dicot foliage consumption appears to result in high Ca isotope values, roots and stems display lower Ca isotope values than foliage and grass (e.g. [24,55]). The unknown macropod has slightly lower Ca isotopic composition than that of the modern wombat. While the data cannot be compared to previous dietary reconstruction as the genus of this macropod is unknown, a mixed or grazing diet could be suggested.

Overall, the Ca isotope composition of some taxa (i.e. *Procoptodon*, *Diprotodon*, and *Macropus*) are consistent with previous dietary reconstructions. Others provide new insights into diets of Pleistocene fauna: *Protemnodon* at Bingara and Wellington Caves may have been consuming monocots or other plant tissues with depleted Ca isotope compositions, and *Petrogale*, unlike modern rock wallabies, may have had a diet dominated by grass. The distinct Ca isotope values of Pleistocene marsupial herbivores at Wellington Caves and Bingara support niche differentiation similar to that observed from C and O isotope studies on Australian herbivore marsupials during Pleistocene climatic fluctuations [5,6,9,12]. The contrast in Ca isotope values between the two large-bodied macropods, *Procoptodon* (high *δ*^44/42^Ca) and *Protemnodon* (low δ^44/42^Ca), and between the two small-bodied macropods, i.e. *Aepyprymnus* (high *δ*^44/42^Ca) and *Petrogale* (low δ^44/42^Ca), could indicate that macropods in the same size classes had distinct Ca isotope niches, potentially corresponding to dietary niches [60].

The Ca isotope composition of Pleistocene marsupial herbivores in this study are narrow when compared to the Ca isotope values found in a modern sub-population of Tasmanian bare-nosed wombats [30]. As the Ca isotope values do not significantly differ between taxa, it cannot be excluded that the smaller ranges of Ca isotope compositions of the taxa are the result of the sampling effect. Therefore, in order to comprehend how potentially different diets of taxa could be reflected in different Ca isotope composition, further research is required to include more individuals from each taxon. In addition, further interpretation will require an examination of plants that were available to the Pleistocene fauna. Such research could record the Ca isotope composition of fossil botanical remains and should map the Ca isotope composition of different plant parts in modern local vegetation (e.g. similar to [61]). Finally, future research could combine environmental and dietary reconstructions of sites and taxa through time to explore hypotheses on dietary changes in response to environmental change (e.g. similar to [5]).

### 4.4. Calcium isotope ratios reflecting juvenile diet

For a given taxon, most early forming teeth have lower Ca isotope compositions than late-forming teeth, with Δ^44/42^Ca_early-late_ < 0 (figure 4). A similar trend has been observed in placental mammals, which is explained by milk consumption during early life and the low δ^44/42^Ca values of milk (e.g. [27–29,62]). Increase in Ca isotope values between early- and late-forming teeth of given individuals in this study may similarly reflect weaning, i.e. the transition between maternal milk consumption in early life to an adult diet consisting of solid food.

The two small-bodied macropods examined here, *Aepyprymnus* and *Petrogale*, both have modern relatives. While tooth eruption timing of modern *Aepyprymnus* has not been documented, M1 eruption in *Petrogale assimilis* has been reported between 226 and 268 days [63], while weaning in this genus occurs on average around 300 days [64]. In the fossil teeth examined here, both *Aepyprymnus* and *Petrogale* show increase in *δ*^44/42^Ca between early and late-forming teeth, with Δ^44/42^Ca <0 (see figure 4*a,b*). Several individuals among *Aepyprymnus* and *Petrogale* display lower Ca isotope compositions in early-forming teeth than in late-forming teeth (i.e. *Aepyprymnus* 47148-1, -3 and -4, and *Petrogale* 47030-6). However, specimen *Petrogale* 47030-3 has a M2 with values similar to those of late-forming teeth. In the same individuals, the low Ca isotope value of an unerupted P3 (figure 4*b*) cannot be explained, as discrimination of heavy isotopes during mineralization would suggest higher Ca isotope compositions in newly forming teeth compared to fully mineralized teeth. Diagenesis owing to porosity when teeth have not fully crystallized would lead to higher Ca isotope values as well [65], as sediment values are high and would overprint lower biogenic signatures [15]. Thus, the Ca isotope composition in this tooth may be an outlier and requires further investigation, beyond the scope of the present study.

For the two large-bodied macropod taxa, *Procoptodon* and *Protemnodon*, both extinct genera, tooth eruption sequences and age of specific tooth formation is unknown. In one of the largest extent macropod species, the eastern grey kangaroo—or *Macropus giganticus*, M1 eruption occurs on average around 370 days, weaning occurs on averaged around 460 days, and M3 erupts around 610 days [64]. It is assumed here that macropods with larger body mass than modern *M. rufus* would have the same or even later molar eruptions. Indeed, in both large-bodied taxa Δ^44/42^Ca < 0, indicate an increase in average Ca isotope compositions. Within *Procoptodon* 47053-1 and *Protemnodon* 126427-1 this is indeed visible; however, *Procoptodon* 106095-1 and *Protemnodon* 113133-1 have relatively high Ca isotope values among early-forming teeth (figure 4). One *Procoptodon* individual (106152-1) with particularly high Ca isotope values in M1 and P3 (figure 4*c*) may have been weaned before or during the formation of M1—such a pattern as previously been observed in deer [29]. Alternatively, this individual may have consumed milk with a high Ca isotope composition: both M1 and P3 might reflect suckling. Unfortunately, as consecutive teeth of the eruptive sequence (i.e. M3 and M4) are missing, it cannot be tested whether they had an even higher δ^44/42^Ca_SRM915a_ than these early-forming teeth (i.e. ≥0.3‰), possibly reflecting a ^42^Ca-depleted adult diet.

Overall, a weaning signal appears to be preserved, similar to placental mammals. Variation of Δ^44/42^Ca_early-late_ within and between taxa could be owing to physiological inter-individual variations of tooth formation and weaning, or variation within and between adult diets. Considerable variation in lactation and tooth eruption (and possibly enamel formation) has been observed in modern macropods (electronic supplementary material, §A) [42,44], and may also have contributed to the observed variation in potential weaning signals observed here.

Variation in weaning does not appear to be taxon-specific, as both patterns (of low and high *δ*^44/42^Ca values in early-forming teeth) are observed in each taxon. Despite this inter-individual variation, the consistency of Δ^44/42^Ca_early-late_ < 0 for each taxon and almost every individual supports the hypothesis that the Ca isotope composition of marsupial teeth generally reflect the transition between milk consumption to an adult diet. However, owing to the low number of individuals that display this pattern, especially among the larger macropods (i.e. *Procoptodon* and *Protemnodon*), further research needs to be conducted that considers the physiological differences of marsupials compared to placental mammals. Marsupial reproductive physiologies are different from those of placental mammals and frequently includes simultaneous gestation and lactation [44], which could alter Ca isotopic compositions of marsupial tissues in distinct ways. Recent studies have shown that high-resolution Ca isotope analysis of various tissues of modern cervids and suids identified different ecological and physiological influences during gestation and lactation, as well as species-specific developments, e.g. antlerogenesis [28,29]. Similar high-resolution marsupial Ca isotopes can shed light on changes of Ca in marsupial life history. [28,29]

The data presented here support the hypothesis that the Ca isotope composition of marsupial dental enamel can be used to study weaning behaviour in extinct marsupials. Our preliminary data show weaning signals consistent with early- and late-forming teeth in both small-bodied and large-bodied macropods. We observe no significant differences in weaning behaviour relative to tooth formation between taxa, nor between large-bodied and small-bodied herbivore marsupials. When considering general patterns of delayed life history, during which tooth formation and eruption in large-bodied macropods occurs at a slower pace than in small-bodied macropods (e.g. [44,64]; see the electronic supplementary material for further details), weaning would have occurred in similar slower rates in mega marsupials. Therefore, if the sample set in this study is representative and there was indeed no difference in relative timing of weaning compared to tooth eruption and life history, this would indicate that in terms of absolute chronology (i.e. actual days or months), mega marsupials would have been weaned much later than the small-bodied taxa.

## 5. Conclusion

Trace elements and REE concentrations of fossil enamel show that the specimens studied here have not been significantly affected by diagenesis, thus allowing the use of Sr and Ca isotopes to reconstruct diet and home ranges of Australian Pleistocene fauna. Consistent Sr ratios across taxa at each site suggest small home ranges for a variety of large- and small-bodied herbivores. This does not support large-scale migration of megafauna and may be indicative of comparatively favourable local environments with rich ecosystems, supporting a diversity of dietary niches.

Variation in Ca isotopes between taxa can be interpreted as distinct herbivorous dietary niches. For some taxa (i.e. *Aepyprymnus*, *Diprotodon*, *Macropus* and *Procoptodon*) Ca isotope compositions agree with previous dietary reconstructions based on dental microwear and C and O isotopes. The Ca isotope composition of *Aepyprymnus* is consistent with a browser's diet, while Ca isotope values in *Macropus* indicate grazing—both are similar to the diet of their modern equivalent species. Ca isotope values of *Diprotodon* suggest mixed feeding behaviour. The Ca isotope composition of *Procoptodon* are consistent with a specialized diet of dicot vegetation. In other taxa (i.e. *Petrogale* and *Protemnodon*), Ca isotope compositions provide new insights into their Pleistocene ecology. In contrast with modern rock wallabies, Pleistocene *Petrogale* may have fed on grass. The low Ca composition of *Protemnodon* could indicate consumption of grasses (monocots), or specific organs of dicot plans (e.g. stems or bark). Taxon-specific variation of Ca compositions points to niche differentiation across Australian Pleistocene herbivores.

Intra-individual Ca isotope variation generally shows a consistent increase in Ca isotope composition with tooth eruption and formation. This suggests that the Ca isotope composition of marsupial teeth can reflect weaning behaviour, as previously observed in placental mammals. Variation between individuals may be caused by different timing of weaning or tooth formation, or different Ca isotope composition of maternal milk owing to variation in adult diets. Similar timing of weaning relative to tooth eruption and formation in the studied large- and small-bodied taxa might show that in absolute chronology, weaning periods of megafauna were later or prolonged.

The combination of Ca and Sr isotopes in fossil tooth enamel suggests that Pleistocene faunal communities of Bingara and Wellington Caves roamed small areas in rich ecosystems that could sustain a diversity of dietary niches. Further food web reconstructions in different environments and geological periods could reveal how these ecological communities adapted to changes in climate and predation pressures. Variation in Ca isotope compositions of different herbivores provide a baseline of distinct food resources for carnivores. This baseline could aid dietary reconstructions of Pleistocene predators, such as *Thylacoleo carnifex,* and identify specialized hunting behaviours that target groups of prey with distinct Ca isotope composition, e.g. juveniles. Such feeding relationship reconstructions will allow testing of hypotheses on causes of megafauna extinction, including juvenile overkill and trophic cascade collapse.

## Data Availability

All data are provided in the electronic supplementary material [67].
